# Crystal Mat Gold

**Published:** 1893-02

**Authors:** Edward C. Kirk


### CRYSTAL MAT GOLD.[1]

BY EDWARD C. KIRK, D.D.S.

At the last meeting I presented the result of an experiment in the use of crystal mat gold, for the reason that since I commenced the practice of dentistry I have experimented from time to time with various forms of sponge gold and have never attained results which were satisfactory until recently. I have taken it up and abandoned it again and again. I have given it limited use, but for such operations as I exhibited at the last meeting I have found it uniformly good. I brought the patient here, and exhibited the fillings, because, to me, they seemed satisfactory; and I asked at that time to have a committee appointed, so that I might have the benefit of their judgment whether they would be accepted as good operations in gold,—regardless of the kind of gold, but simply as gold fillings.

I now wish to state what I have to say in regard to the method which I employed in those operations.

The principal difficulties which I have heretofore experienced in the use of any of the well-known forms of sponge or crystal gold have been the inability to secure satisfactory margins, and the tendency which the material has of balling up, and so bridging unfilled spaces in the cavity, and at the same time producing an unevenness of surface to the gold plug which it is difficult to counteract. Heretofore the amount of delicate manipulation and careful work necessary to insert a filling of sponge gold which would compare favorably with fillings of foil has been so great that the amount of time consumed in making such a filling constituted the greatest obstacle in its employment. There seems to be no doubt that a filling can be made of sponge gold which answers all the requirements of a perfect filling as satisfactorily as one made of foil, but for the reasons noted, and under former conditions, it seemed to bear no comparison to gold-foil in ease of manipulation and the consequent amount of time necessary for its insertion. Some experiments recently undertaken, with the view of determining the adaptability and comparative working-qualities of the form of sponge gold known as crystal mat, have brought out certain new data with regard

[1] Read at a meeting of the Odontological Society of Pennsylvania, December 10, 1892.

to the working-qualities of this kind of gold which lead me to believe that when its manipulation is properly understood and definitely carried out in harmony with certain principles, its introduction can be accomplished with a degree of certainty, ease, and rapidity equalling, if not surpassing, that of foil and its various preparations. First, with regard to the marginal preparation of cavities. In order to insure the best results with crystal mat the margins of cavities must be strong, and formed with a bevel. They must present a square shoulder, so that when the gold is finally finished it will be butted up against the margin without any overlap. Where a bevel is used I have found it next to impossible to prevent the wedge-shaped edge of gold which forms the slight overhang at the bevelled margin from breaking in finishing, but if the margins are made square-shouldered, as stated, this does not occur, and the joint can be made absolutely perfect with ordinary care. The use of sponge gold in starting fillings by filling undercuts or retaining-points is well known, and its use for this purpose has gained wide acceptance by the best operators. It is therefore not necessary to comment upon the value of this kind of material for that purpose. In the filling of the body of the cavity, after the anchorages are disposed of, I have found the best results to follow the method which I shall now describe. A pellet of crystal mat gold approximating the size and shape of the floor of the cavity should be selected, and, after annealing, carefully placed in position, so that it well covers the floor and is in contact with the anchorages, care being taken to introduce it with as little compression as possible. A plugger point with shallow serrations is then used to tack it to the anchorages, after which a broad-faced foot-plugger, serrated in but one direction preferably, is used to compress the pellet into place by hand-pressure. When it has been firmly condensed by hand-pressure it should be malleted into a uniformly homogeneous mass.

A number of operators have expressed the opinion that crystal gold should not be densely malleted, claiming that better results are obtained by the use of a light mallet-blow, or by hand-pressure. My experience leads me to take exception to this. I know of no form of gold that will endure the amount of malleting which crystal mat will. It becomes denser and more homogeneous by hard malleting than any form of gold with which I am acquainted. This seems to be born out by a study of the physical make-up of crystal mat gold as compared with foil. In the use of foil the amount of force necessary to bring about perfect cohesion of superimposed laminæ is inconsiderable. This is shown by the ease with which solid plugs can be made of foil by hand-pressure or by the rotation method; but with crystal mat, or any form of sponge gold, continuous and evenly-applied malleting is necessary to bring into perfect cohesion the minute particles of gold which constitute its make-up. The malleting must proceed in every direction, in order that the plug shall be of uniform density and of perfect cohesion throughout, a result which cannot be accomplished by ordinary hand-pressure. The principle which should govern in the condensation of crystal mat gold is to first bring about a moderate cohesion of its particles by means of hand-pressure, whereby it is made more strongly coherent, and is thus prepared for the further condensing action of the mallet. If the mallet were applied from the first to the soft, loosely-held-together piece of sponge gold, the effect would be to disintegrate it, or break it up into particles; but if partial condensation is first secured by hand-pressure, the cohesion of the mass is sufficiently great to withstand any amount of malleting, which only serves to further condense and solidify it.

Before dismissing the question of margins I wish to allude to a device which I have used to secure perfect marginal finish where the cavity edges have been prepared otherwise than square, as before advocated. When the cavity is filled flush with its margins with crystal mat, thoroughly condensed, the margins can be made perfect by malleting on several thicknesses (three or four) of No. 20 rolled gold to perfect the margin, and then continuing with crystal mat until contour is fully restored. The points with which I have secured the best results are the finer numbers, extremely small, with shallow serrations. My habit with foil fillings is to use a rather broad surface-plugger, but I have found better results in using crystal mat with the fine points, as noted. The cohesive quality of crystal mat is quite remarkable. In several instances where I have found imperfections of margins on the labial aspect of approximal incisor fillings, a defect, for instance, which had developed under the use of the plug-finishing burr, I have been able to add to the surface a sufficient quantity of crystal mat gold to restore the imperfection without making any grooves or retaining-points in the gold. In other words, the surface of a plug made with crystal mat gold which has been cut by a plug-finishing burr is sufficiently cohesive to retain additions of cohesive gold to its surface and form a solid filling without undercutting or grooving the plug for the purpose of gaining a retaining hold. The point at which condensation has been perfectly performed is well indicated in the color of the surface, which changes under the mallet as the condensation progresses. By constant malleting the brown tint of the crystal mat gold disappears, and its color becomes lighter and lighter, until it is in no way distinguishable from that of a foil filling. If, during the process of malleting, the surface of the gold plug be examined from time to time with the glass, it will be seen to present areas which are perfectly bright and metallic-looking, and of the normal gold-color, and between these, slightly darker and brownish particles, which represent the non-condensed particles of crystal gold. The malleting should be continued until all of these brownish specks or particles disappear and the surface of the plug presents a uniformly golden-yellow appearance and solid metallic texture. An extremely valuable use which I have made of crystal mat gold is in connection with an oxyphosphate of zinc lining in cavities of extensive decay, where the walls are so weakened or imperfect that further cutting, for the purpose of securing anchorage, becomes undesirable, as producing an element of weakness in the operation. In these cases I have adopted a plan which is a modification of a method suggested by Dr. Oltramare, in a paper before the last Swiss Congress, as follows: The cavity is prepared with as much anchorage in the tooth-structure as is obtainable, consistent with strength, and then lined with a small amount of oxyphosphate of zinc in a rather soft, sticky condition. A pellet of crystal mat gold, approximating the form of the floor of the cavity, is embedded for about half its depth in the cement, and time given for the cement to thoroughly harden. All excess of cement is then removed with an excavator, and the crystal mat gold is afterwards condensed and malleted strongly to place. This furnishes a cohesive foundation, upon which the balance of the filling may be applied, in connection with such retaining anchorages as it has been otherwise possible to secure.

A large number of fillings inserted in this manner during the past year have been extremely satisfactory, and show no evidence thus far of any tendency to leak or become loose. The rapidity with which a filling may be made with crystal mat gold is somewhat remarkable. I have made no accurate time observations in comparison with foil, so that I cannot give exact data in regard to it; but I am quite sure that the time consumed in the filling of a cavity, by the method described, with crystal mat gold is from a third to a half less than would be required to do the same operation with foil. Its great value, so far as rapidity is concerned, is demonstrated where extensive contours are to be made. It is not my intention to advocate a wholesale use of this material, to the exclusion of other forms of gold. My object in calling attention to it in the manner here described is to emphasize what I believe to be a fact,—that perfect fillings can be made with this material, in certain classes of cavities, with more ease and rapidity, and with as good, if not better, results than with foil. The objections which have been raised to the extensive use of the ordinary sponge, or crystal forms of gold, are no doubt just, and have had their foundation in several causes, which, in the form of sponge gold under consideration, are, I believe, obviated. In the first place, the preparation is absolutely pure gold. At the time sponge, or crystal, gold was first introduced to the notice of the profession, or soon after, a number of processes for making this material were published, and the consequence was that many dentists made it for themselves, or its manufacture was lodged in unskilled hands, with the result that the product was more or less contaminated with substances which acted injuriously upon the tooth, or in some way affected the integrity of the filling. More than this, the crystal form of the product was not uniform, and was so variable in the shape and size of its ultimate particles that no two samples acted or worked in exactly the same way; hence, great variety in results was the natural consequence. Again, it was put upon the market in large blocks, and it was left to the operator to cut or tear apart, getting pieces of irregular size and form, which introduced a feature of inconvenience in working-quality, resulting in irregular condensation of the mass, unevenness of the filling, pitting, etc. Such gold was known to ball up, or bridge over, and produce a plug which lacked homogeneity. Crystal mat, besides being an absolutely pure product and free from the chemical impurities which characterized the earlier preparations of sponge gold, is furnished by the manufacturer in pellets of assorted sizes, so that the operator has ready at hand a range of sizes from which to select the pellets for filling a given cavity, and with these at hand, and a uniform mallet-power, the work proceeds evenly throughout, resulting in a perfectly homogeneous plug. In conclusion, the best results with this material, I believe, are obtainable only with the Bonwill mechanical mallet, with a spring-roller cam. The character of the blow delivered by this instrument, properly adjusted, produces a result which I firmly believe is not otherwise obtainable.

